# Lactate-induced M2 polarization of tumor-associated macrophages promotes the invasion of pituitary adenoma by secreting CCL17

**DOI:** 10.7150/thno.53749

**Published:** 2021-02-06

**Authors:** Anke Zhang, Yuanzhi Xu, Houshi Xu, Jie Ren, Tong Meng, Yunjia Ni, Qingwei Zhu, Wen-Bo Zhang, Yuan-Bo Pan, Jiali Jin, Yunke Bi, Zhe Bao Wu, Shaojian Lin, Meiqing Lou

**Affiliations:** 1 Department of Neurosurgery, Shanghai General Hospital, Shanghai Jiao Tong University School of Medicine, Shanghai, China.; 2 Department of Neurosurgery, Center of Pituitary Tumor, Ruijin Hospital, Shanghai Jiao Tong University School of Medicine, Shanghai, China.; 3 Division of Spine, Department of Orthopedics, Tongji Hospital affiliated to Tongji University School of Medicine, Shanghai, China.; 4 Department of Neurosurgery, The Children's Hospital of Zhejiang University School of Medicine, National Clinical Research Center for Child Health, Hangzhou, Zhejiang, China.; 5 Department of Neurosurgery, Second Affiliated Hospital, School of Medicine, Zhejiang University, Zhejiang, China.; 6 Department of Central Laboratory, Shanghai Tenth People's Hospital of Tongji University, School of Medicine, Tongji University, Shanghai, China.

**Keywords:** pituitary adenoma, macrophages, CCL17, lactate acid, mTOR

## Abstract

**Background:** Lactate greatly contributes to the regulation of intracellular communication within the tumor microenvironment (TME). However, the role of lactate in pituitary adenoma (PA) invasion is unclear. In this study, we aimed to clarify the effects of lactate on the TME and the effects of TME on PA invasion.

**Methods:** To explore the correlation between TME acidosis and tumor invasion, LDHA and LAMP2 expression levels were quantified in invasive (n = 32) and noninvasive (n = 32) PA samples. The correlation between immune cell infiltration and tumor invasion was evaluated in 64 PAs. Critical chemokine and key signaling pathway components were detected by qPCR, Western blotting, siRNA knockdown, and specific inhibitors. The functional consequences of CCR4 signaling inhibition were evaluated *in vitro* and *in vivo*.

**Results:** Lactate was positively associated with PA invasion. Of the 64 PA tissues, invasive PAs were related to high infiltration of M2-like tumor-associated macrophages (TAMs) (P < 0.05). Moreover, lactate secreted from PA cells facilitated M2 polarization *via* the mTORC2 and ERK signaling pathways, while activated TAMs secreted CCL17 to promote PA invasion *via* the CCL17/CCR4/mTORC1 axis. According to univariate analysis of clinical data, high CCL17 expression was associated with larger tumor size (P = 0.0438), greater invasion (P = 0.0334), and higher susceptibility to postoperative recurrence (P = 0.0195) in human PAs.

**Conclusion:** This study illustrates the dynamics between PA cells and immune TME in promoting PA invasion *via* M2 polarization. CCL17 levels in the TME are related to the PA invasiveness and clinical prognosis, and the CCL17/CCR4/mTOCR1 axis may serve as potential therapeutic targets for Pas.

## Introduction

Pituitary adenomas (PAs) represent approximately 10% of intracranial tumors 1. Most PAs are noninvasive, following a slow growth pattern and remaining within the sella and/or displacing the surrounding tissues. However, up to 25-55% of PAs may exhibit infiltrative characteristics (Knosp IIIb and IV), namely, invasion of the cavernous sinuses and sphenoid sinus as well as focal or extensive bone erosion; these tumors are regarded as invasive PAs 2. Invasive PAs have significantly higher proliferation rates and poorer prognosis than noninvasive PAs 2. Compared with patients with noninvasive adenomas, patients with invasive adenomas have a significantly lower 6-year survival rate postsurgery. Therefore, exploring the mechanism underlying PA invasion is necessary for improving its diagnosis and treatment.

Tumor invasion is highly associated with the tumor microenvironment (TME) 3. One of the driving forces that shift the TME toward a hostile milieu for antitumor immune cells is hypoxia resulting in a cascade of biochemical reactions leading to localized acidification. Lactate has been reported to be a signaling molecule that plays an important role regulating metabolic pathways, immune responses, and intracellular communication within the TME 4-6. In addition, lactate is found at high concentrations within the anaerobic tumor environment and has been shown to alter macrophages to acquire properties that enhance tumor growth 7. Tumor-associated macrophages (TAMs) are involved in homeostatic functions that promote malignant tumor initiation and progression 8, 9. Several studies have shown that a small number of macrophages are observed in all types of PAs 10-12; however, the role of TAMs in PA invasiveness is still unknown.

TAMs release a variety of anti-inflammatory cytokines (*e.g.*, IL10 and TGFB1) and chemokines (*e.g.*, CCL17, CCL18, and CCL22) that inhibit dendritic cell maturation, which limits antigen presentation and favors the recruitment of immunosuppressive Treg cells 13, 14. It has been demonstrated that CCL17-expressing CD163^+^ M2-like macrophages promote tumor metastasis in nonsmoking patients with lung adenocarcinoma 15. In addition, high levels of intratumoral CCL17 expression are significantly associated with aggressive clinical pathological characteristics and poor overall survival in HCC patients 16. The CCL17/C-C chemokine receptor 4 (CCR4) axis has been recently reported to induce the migration of hepatocellular carcinoma cells 17 and enhance epithelial-mesenchymal transition (EMT) in breast cancer *via* Akt signaling 18. However, there are few studies detailing intracellular communication and TME remodeling in PAs.

In this study, we described for the first time the correlation of tumor invasiveness of PAs with the immune TME. Lactate secreted from invasive PAs can facilitate the differentiation of TAMs towards an M2-like phenotype. Moreover, CCL17 released from M2-like TAMs enhances tumor invasion of PAs *via* the CCL17/CCR4/mTORC1 axis. Finally, CCL17 is highly correlated with PA invasiveness and a higher Knosp grade.

## Materials and Methods

### Clinical data and human tissues samples

This study was conducted in accordance with the Declaration of Helsinki. Human PAs tissues were considered exempt by the Human Investigation Ethical Committee of Shanghai General Hospital affiliated to Shanghai Jiao Tong University. Human PA samples were consecutively collected between January 2015 and January 2019 from the Department of Neurosurgery in Shanghai General Hospital. A total of 64 patients with PAs underwent surgery using the endoscopic endonasal transsphenoidal approach for the first time and had not previously received radiotherapy or chemotherapy. Clinical definition of invasive PAs was diagnosed according to Knosp classification, in which Knosp III and IV are considered invasive tumors 19. Pathological diagnosis was based on the 2017 World Health Organization (WHO) classification of tumors of the pituitary gland using the following formula: ABC/2; where A is the maximum tumor diameter; B is the diameter of the tumor perpendicular to A; and C is maximum height of the tumors as reported on the MRI scan. All samples were obtained from patients who signed informed consent approving the use of their tissues for research purposes after the operation.

### Immunohistochemistry

Patient tumor samples and animal tumor tissues were fixed in 4% paraformaldehyde for 24 h and then embedded in paraffin. Paraffin blocks were cut into 5 μm sections and used for hematoxylin and eosin (HE) or immunohistochemistry staining. For immunohistochemistry (IHC), rehydrated tissue sections were blocked with goat bovine serum overnight at 4 °C and then were stained with CD68 (catalog no. ab125212; clone, polyclone; 1:200; Abcam), CD4 (catalog no. ab133616; clone, EPR6855, 1:200, Abcam), CD8 (catalog no. ab93278, clone, EP1150Y; 1:200; Abcam), MPO (catalog no. ab134132, clone, EPR4793, 1:200, Abcam). After washing, the sections were incubated with biotinylated anti-mouse IgG or biotinylated anti-rabbit IgG (Vector Laboratories, CA, USA). For dectection, the ABC method (Vector Laboratories) was used with 3,3' diaminobenzidine (Dojindo Laboratories, Kumamoto, Japan) as a substrate. The sections were observed using an AX-80 microscope (Olympus, Tokyo, Japan). Images were analyzed using Image J software (http://imagej.nih.gov/ij/) and quantification of positive expression was calculated. For statistical analysis, the Fisher's exact test and Spearman's rank correlation coefficient were used, and P values less than 0.05 were considered statistically significant.

### Immunofluorescence staining

Cells were washed with PBS and fixed in an ice-cold acetone-methanol mixture (1:1) for 5 min. After washing in PBS 3 times, the cells were incubated in a blocking buffer (PBS with 3% donkey serum and 0.3% Triton X-100, pH 7.4) at room temperature for 2 h. Cells were then stained with primary antibody against CD68 (catalog no. ab31630, 1:100, Abcam) or CD301 (catalog no. ab77398, 1:100, Abcam) with 5% normal goat serum in PBS at 4 °C overnight. A secondary antibody conjugated to Alexa Fluor 594 or 488 (catalog no. A-21207 or A-11055, 1:250, Life Technologies) was used to visualize the primary antibody and cells were counterstained with 4,6-diamidino-2-phenylindole dihydrochloride (DAPI).

For immunofluorescent staining of human tissue samples, tumor tissues or pituitary tissues were fixed in 4% paraformaldehyde for 24 h and then embedded in paraffin. Paraffin blocks were cut into 5 μm sections. After washing in PBS 3 times, sections were incubated in a blocking solution at room temperature for 2 h. Samples were stained with primary antibodies against LDHA (catalog no. ab125683, 1:200, Abcam), LAMP2 (catalog no. ab199947, 1:200, Abcam), β-Actin (catalog no. ab8226, 1:200, Abcam), or CCL17 (catalog no. ab182793; 1:100; Abcam), in an antibody reaction buffer (1% BSA and 0.3% Triton X-100 in PBS) overnight at 4 °C. Samples were then incubated with secondary antibodies conjugated with Alexa Flour 488 or 594 (catalog no. A-21206 or A-21207, 1:250, Life Technologies) at room temperature for 1 h, and DAPI was used for nuclear staining. Images were visualized and photographed using a fluorescence microscope (IX 71, Olympus, Tokyo, Japan).

### Cell culture and reagents

The GH3, MMQ, and THP1 cell lines were purchased from the American Type Culture Collection (ATCC, Manassas, VA, USA). GH3 and MMQ cells were cultured in DMEM/F12 medium (Hyclone, UT, USA) supplemented with 2.5% FBS (Invitrogen, CA, USA), 15% horse serum (Invitrogen) and 1% antibiotic mixture. THP1 cells were cultured in RPMI 1640 complete medium. For cytokine stimulation and inhibitor-based treatment experiments, cells were serum starved for 2 h and then treated under the indicated conditions.

Chemical compounds used in this study included DMSO (D2650, Sigma-Aldrich, St. Louis, MO, USA), AZD2098 (HY-U00064, MedChem Express, NJ, USA), and L-(+)-Lactic acid (L6402, Sigma-Aldrich, St. Louis, MO, USA). CellTiter-Glo® Luminescent Viability Assay reagent was purchased from Promega (Madison, WI).

### Collection of conditioned medium (CM)

GH3 and MMQ cells were plated in 6-well plates at a concentration of 5×10^5^, 1×10^6^, and 2×10^6^ cells, and the supernatants were collected as CM1, CM2, and CM3, respectively, after 24 h. Primary human PA cells were isolated from tumor samples after surgical resection and plated in 6-well plates at 2×10^6^ cells. After 24 h, the supernatant was collected as human PA-derived CM.

### Isolation of rat bone marrow-derived macrophages

Rat bone marrow (BM) was surgically dissected from the thigh bone and tibia of Wistar Furth rats and mechanically separated into single cells by vigorous pipetting. Isolated BM was cultured in RPMI 1640 medium with 10% FBS, rMCSF (10 ng/mL) and 1% penicillin-streptomycin at 37 °C and 5% CO_2_. After 3 days, the medium was changed and non-adherent cells were removed.

### Cell viability assays

Cell viability was measured in at least three duplicate samples with the CellTiter-Glo® luminescence assay (Promega) according to the manufacturer's protocol in three independent experiments. Cells were seeded in a 96-well plate at a density of 2 ×10^3^ cells per well and treated with indicated doses of macrophage CM with or without rapamycin or CCR4 antagonist. Cells were cultured for 24, 48 and 72 h before adding 100 μL of the CellTiter-Glo® luminescence assay reagent in each well. Cells were incubated for an additional 10 min at room temperature to stabilize luminescent signals and transferred to 96-well black plates. Measurements were performed using a luminescence reader (TECAN, Männedorf, Switzerland).

### Cell cycle distribution analysis

Treated cells were trypsinized and fixed in ice-cold 70% ethanol and incubated overnight at -20 °C. Fixed cells were washed with chilled PBS, stained with triton X-100/PI/RNase solution, and analyzed by a flow cytometer.

### Real-time RT-PCR

Total RNA was extracted from tissue samples and cells using TRIzol reagent (Invitrogen) after washing with PBS. cDNA was synthesized from purified RNA using a SuperScript III First-Strand cDNA synthesis system (18080051, Life Technologies) according to the manufacturer's instructions. SYBR Green PCR Master Mix (Applied Biosystems, CA, USA) was used for PCR amplification and a real-time PCR machine (iQ5, Bio-Rad Laboratories) was used to quantify the expression of mRNAs. β-actin was used as an endogenous control and the expression levels were quantified using 2-∆∆Ct method. All primer sequences are listed in Supplementary Table 1, and detection of each primer was performed in triplicate.

### Western blotting

Cells and tissues were lysed by RIPA buffer (Beyotime Biotechnology, Beijing, China) with protease inhibitor cocktail (B14002, Biotool), and total protein concentration was measured using a bicinchoninic acid protein assay kit (Tiangen Biotech, Beijing, China). Western blotting was performed with primary antibodies against β-actin (catalog no. ab6276; 1:1000; Abcam), phosphorylated p70S6K (Thr389) (catalog no. 9234; 1:1000; CST), p70S6K (catalog no. 2708; 1:1000; CST), phosphorylated 4EBP1 (Thr70; catalog no. 2983; 1:1000; CST), 4EBP1 (catalog no. 9644; 1:1000; CST), phosphorylated Akt (S473;catalog, 4060; 1:1000; CST), Akt (catalog no. 4685; 1:1000; CST), phosphorylated mTOR (catalog no. 2971; 1:1000; CST), mTOR(catalog no. 2983; 1:1000; CST), phosphorylated ERK (catalog no. 4370; 1:1000; CST), ERK (catalog no. 4695; 1:1000; CST), PCNA (catalog no. 13110; 1:1000; CST), vimentin (catalog no. 5741; 1:1000; CST), E-cadherin (catalog no. sc-8426; 1:500; Santa Cruz) and N-cadherin (catalog no. sc-8424; 1:500; Santa Cruz). Western blots shown in the accompanying figures are derived from three independent experiments.

### NOD/SCID tumor-bearing mouse model

NOD/SCID female mice were purchased from Slaccas Company (Shanghai), and they were used at 4-5 weeks of age. Mice were housed and maintained in a specific pathogen-free (SPF) environment. NOD/SCID mice were were injected subcutaneously with 1×10^6^ GH3 cells, or 1×10^6^ GH3 cells mixed with 1×10^6^ M0 macrophages re-suspended in 0.1 ml of PBS. After sacrificing mice, tumor samples were weighed and stored for further studies. Studies with animals were approved by the Institutional Animal Care and Use Committee of Tongji University (KS1607).

### Flow cytometry analysis

Tumor tissues were removed, minced in phosphate-buffered saline (PBS) containing 10 mM CaCl_2_, digested at 37 °C for 150-180 min in DMEM containing 10 mM CaCl_2_ and 1 mg/ml collagenase type I (Sigma-Aldrich, SCR103), filtered through a 70-μm strainer, and centrifuged at 700 g for 5 min. Cells were collected and resuspended in red blood cell lysis buffer (eBioscience RBC Lysis Buffer) before further analysis. To isolate TAMs, pellets were washed twice with PBS and once with FACS staining buffer followed by staining with F4/80 APC (Biolegend, 123115), CD11b PE (Biolegend, 101207), CD11c Percp (Biolegend, 117325), and CD206 FITC(Biolegend, 141703) on ice for 1 h in the dark. After staining, cells were washed twice with staining buffer, suspended in flow cytometry buffer (PBS with 0.5% BSA and 2 mM EDTA), and analyzed using the BD LSRFortessa Cell Analyzer (BD Biosciences). Data were analyzed using FlowJo software version X.0.7 (Tree Star, Inc.).

### Gene set enrichment analysis (GSEA)

We used GSEA to compare normal pituitary and invasive PA (6 aggressive/invasive prolactin pituitary tumors and 5 non-invasive prolactin PAs were extracted from Gene Expression Omnibus, GEO; https://www.ncbi.nlm.nih.gov/geo, GSE22812). GSEA was performed with the WEB-based Gene Set Analysis Toolkit (WebGestalt, http://www.webgestalt.org/).

### Statistical analysis

Statistical analyses were performed using SPSS analysis tools (IBM Corp.) or Prism8 software program (GraphPad Software). All data are presented as mean±SEM. To calculate statistical significance between two groups, a two-tailed unpaired Student's t-test (for parametric analysis) or Mann-Whitney U test (for non-parametric analysis) was performed. One-way analysis of variance (ANOVA) followed by Tukey's or Dunnett's multiple comparisons test was used to detect the differences in the results between groups. Two-way repeated measures ANOVA followed by Bonferroni's multiple comparisons test was used for the proliferation assay. The correlation between the expression profiles of immune biomarkers and tumor invasion of PAs was analyzed using Spearman's rank test. *P* values less than 0.05 were considered statistically significant. No sample outliers were excluded. Individual *in vitro* experiments were performed at least two times with similar results. For *in vivo* experiments, data were collected from multiple independent experiments performed on different days with different mice.

## Results

### Lactate and TME acidification are positively correlated with PA invasion

Lactate has steadily come to be appreciated as an essential component of tumor carcinogenesis 20, yet its intrinsic role and mechanism of action in PA invasiveness remain unknown. It has been demonstated that lactate dehydrogenase (LDH)-mediated excessive production of lactic acid and lysosome-associated membrane protein 2 (LAMP2)-induced TME acidosis favor tumor immunoevasion melanomas 21.

**Figure 1:**
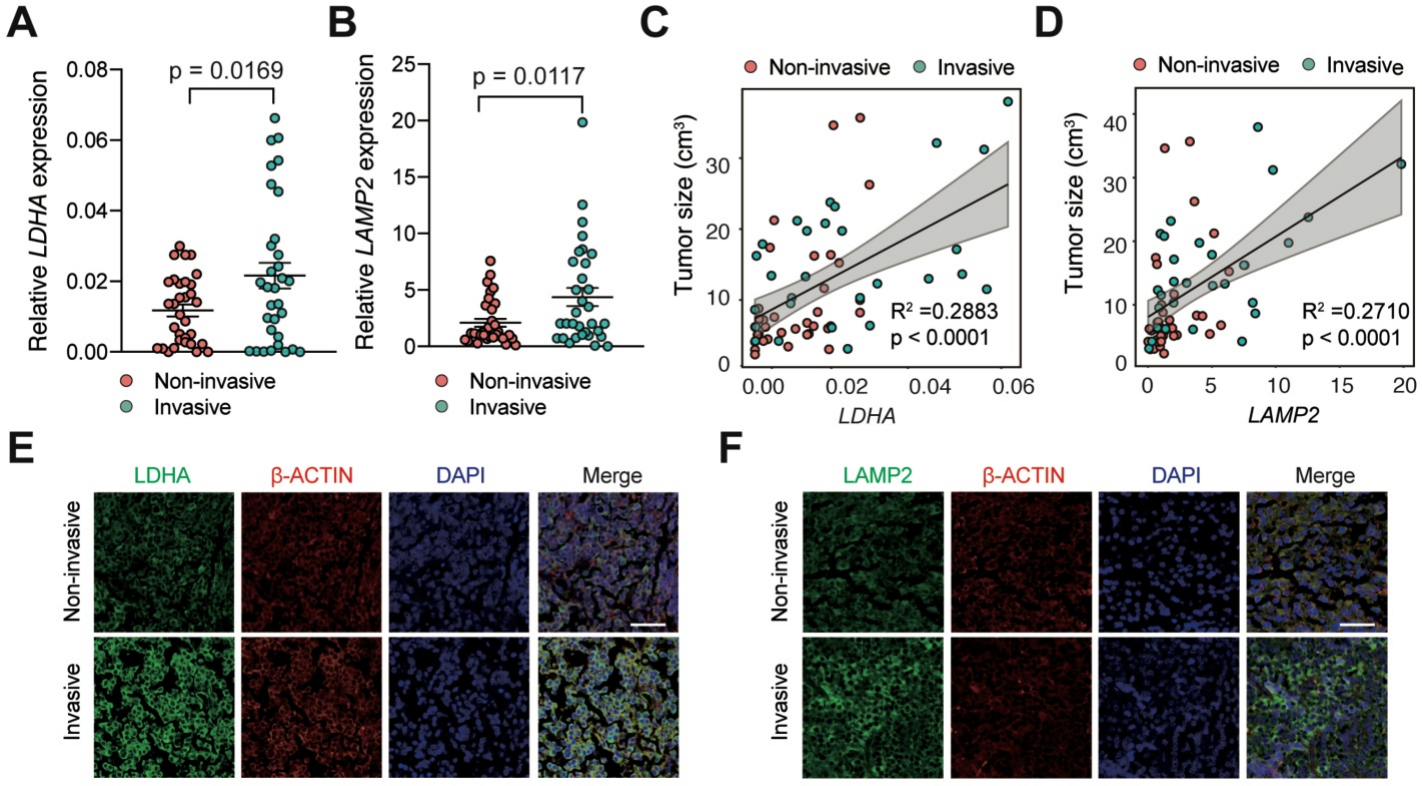
** Lactate and TME acidosis positively correlated with PAs invasion. A and B,** Expression levels of (**A**) *LDHA* and (**B**) *LAMP2* genes in invasive PA samples (n = 32) versus noninvasive samples (n = 32). **C and D**, Correlation of tumor size with (**C**) *LDHA* and (**D**) *LAMP2* expression levels (n = 64). **E and F**, Representative immunofluorescence images of human PA samples co-stained with (**E**) LDHA or (**F**) LAMP2 (green) and β-Actin (red) demonstrated more lactate production and greater TME acidosis in invasive PAs compared with noninvasive PAs. Cell nuclei were counterstained with DAPI. Original magnification, ×200. Scale bar, 50 µm. All t-tests were two-tailed. Mean ± SEM.

To investigate whether lactic acid is responsible for tumor invasion in PAs, we measured the levels of *LDHA* and *LAMP2* expression in noninvasive human PAs (n = 32) by qRT-PCR and compared them with those in invasive PA samples (n = 32). The results showed that *LDHA* and *LAMP2* mRNA expression levels were significantly elevated in invasive human PAs compared with noninvasive PAs (Figure 1A-B, P < 0.05). Moreover, we discovered a strong positive correlation between the expression of lactate-related genes and tumor size (Figure 1C-D). Similarly, IF staining revealed a profound increase in LDHA and LAMP2 expression in invasive PAs compared with noninvasive samples (Figure 1E-F). These findings indicated that the invasive PAs have significantly higher lactic acid levels.

### M2-like TAMs are positively associated with PA invasion

**Figure 2:**
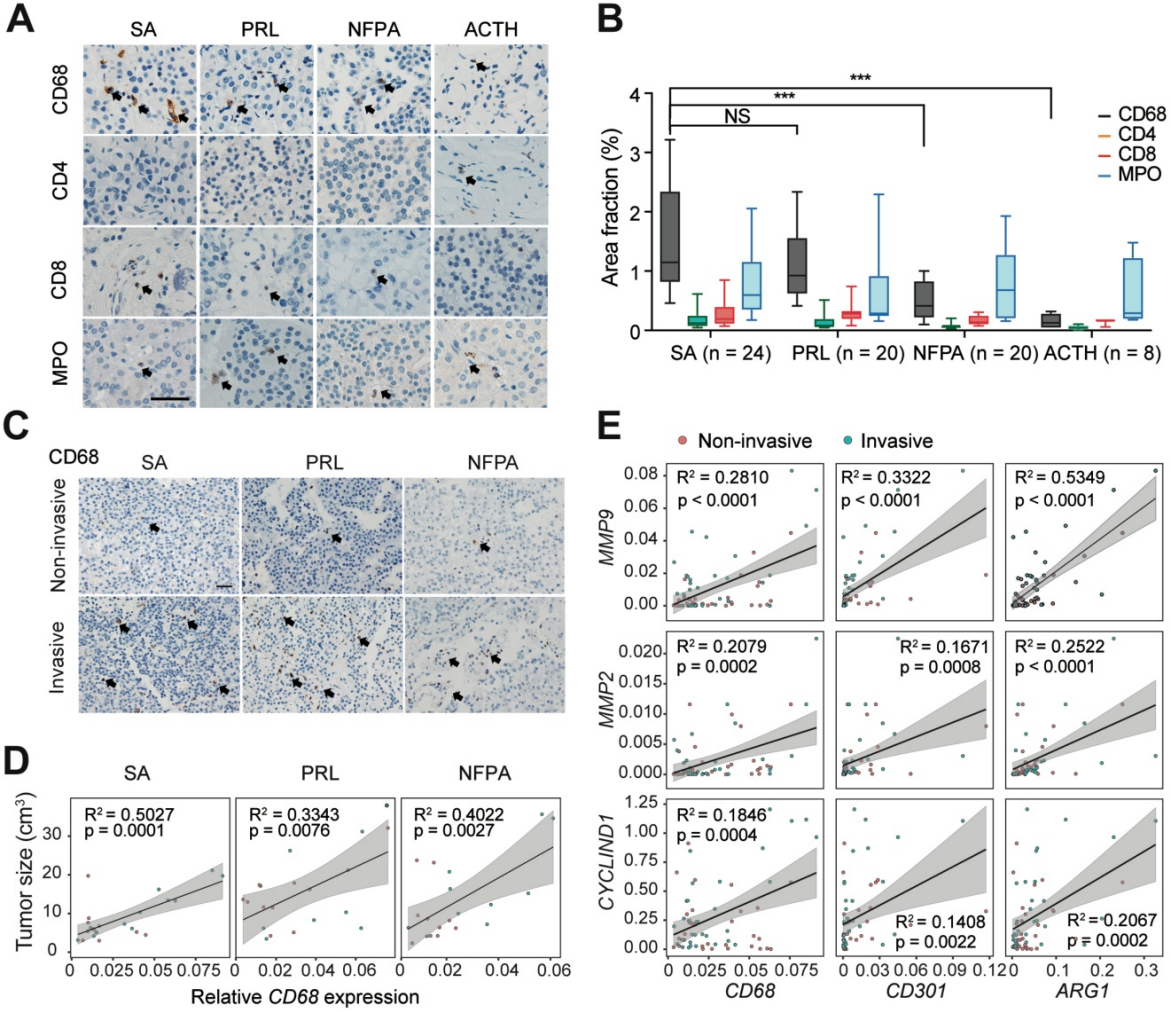
** M2-like TAMs are positively associated with PAs invasion. A and B,** Representative images (**A**) and quantitation of staining percent (**B**) of human SAs, PRLs, NFPAs, and ACTH adenomas stained with CD68, CD4, CD8, and MPO. Scale bar = 50 µm. (SAs, n = 24; PRLs, n = 20; NFPAs, n = 20; and ACTH adenomas, n = 8). Positive cells are indicated with black arrowheads. Original magnification, ×200. Scale bars, 50 µm. Arrow indicates positive cells. **C,** Representative images of SA, PRL, and NFPA samples stained with CD68 in invasive PAs versus noninvasive PAs. Positive cells are indicated with black arrowheads. Original magnification, ×200. Scale bars, 50 µm. **D,** Correlation between *CD68* expression levels and tumor size in human SAs, PRLs, and NFPAs. **E,** mRNA expression of *MMP2*, *MMP9*, and *CyclinD1* correlated with M2-like TAM markers of (*CD68*, *CD301* and *CD206)*. All t-tests were two-tailed. Mean ± SEM. NS, no significance. *P < 0.05, **P < 0.01, and ***P < 0.001. SAs, somatotroph adenomas; PRLs, prolactinomas; NFPAs, non-functioning pituitary adenomas; ACTH adenomas, ACTH secreting adenomas.

It has been demonstrated that tumor invasion is highly associated with the immune TME in PA 22. Thus, we explored the infiltration characteristics of the immune cells in PAs through IHC staining. Notably, we found that CD68^+^ macrophages were more abundant in the PA stroma than CD4^+^ T cells, CD8^+^ T cells, and MPO^+^ neutrophils (Figure 2A). In addition, we observed significantly greater infiltration of macrophages in somatotroph adenomas (SAs) and prolactinomas (PRLs) than in nonfunctioning pituitary adenomas (NFPAs) and adrenocorticotropic hormone (ACTH) adenomas (P < 0.05, Figure 2B). The infiltration of CD68^+^ macrophages was significantly more pronounced in invasive PAs than in noninvasive tumors (Figure 2C). Moreover, there was a positive correlation between macrophage infiltration and tumor sizes in SAs, PRLs, and NFPAs (Fig. 2D); however, there were no such correlations with CD4^+^ T cells, CD8^+^ T cells, or MPO^+^ neutrophils (Figure S1). Furthermore, we observed that M2 macrophage markers (CD68, CD301, and ARG1) were positively correlated with the expression of MMP9, MMP2, and CYCLIND1 in human PAs (Figure 2E), yet the expression of M1 macrophage markers (RANTES, TNF-α, and MCP1) did not show the same trend (Figure S2).These findings indicated that infiltration of M2-like TAMs are positively correlated with tumor invasion in PAs.

### Lactic acid induces the polarization of TAMs toward a M2-like phenotype in PA

Based on the above results, we next investigated whether increased lactate production and increased TME acidosis directly promote PA invasion. We treated GH3 and MMQ cells with 0, 4, 8, and 16 mmol/ml lactic acid. Unexpectedly, the proliferation rates of both cell lines did not differ at the indicated timepoints (Figure S3), which suggested that lactate indirectly exerts tumorigenic effects.

**Figure 3:**
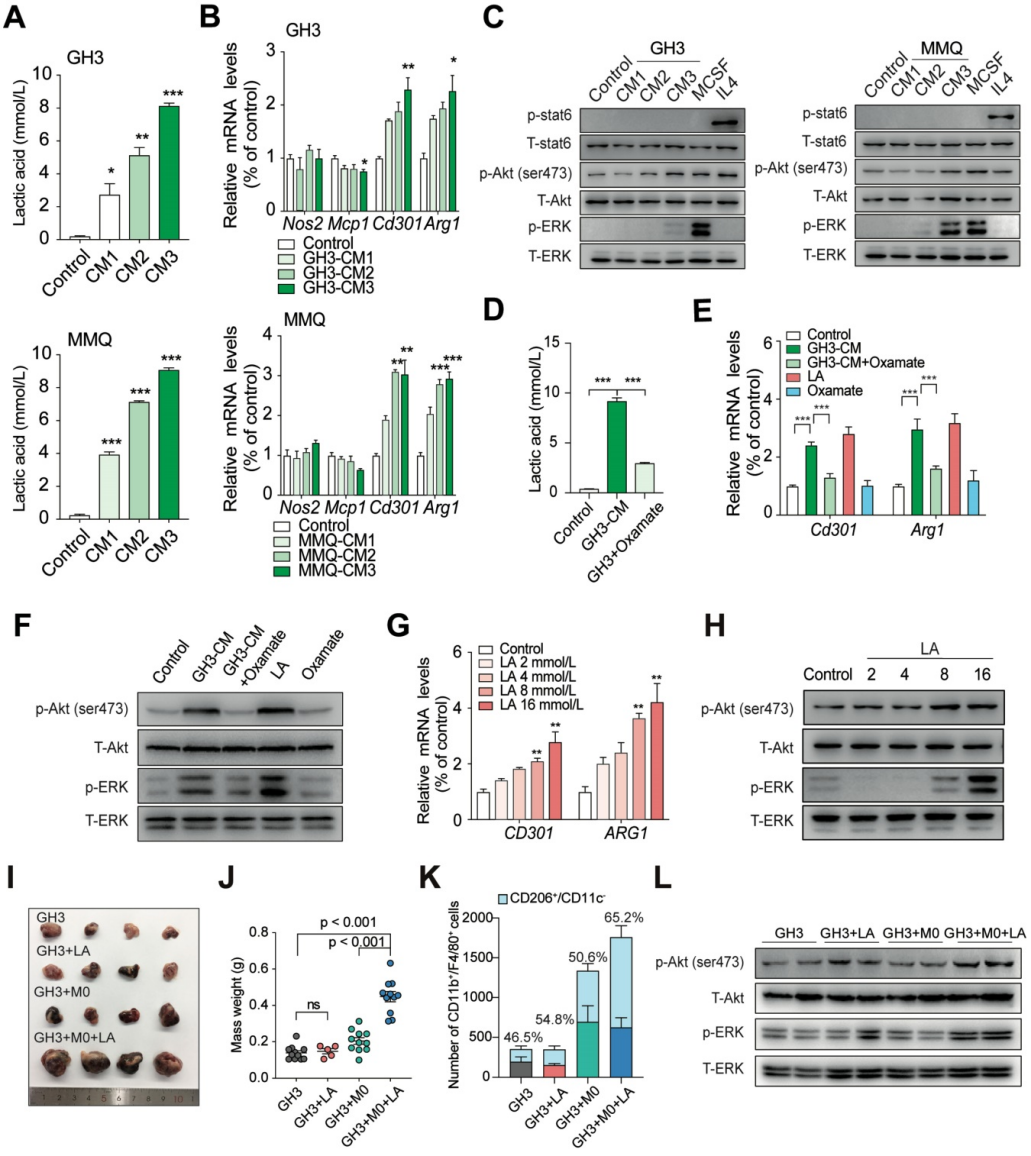
** Lactic acid induces the M2-like polarization of macrophages in PA. A,** Concentration of lactic acid in CM from GH3 or MMQ cells. **B**, Quantification of *Nos2, Mcp1, Cd301* and* Arg1* mRNA expression in BMDMs stimulated with CM from GH3 and MMQ cells for 24 h. **C**, Western blot analysis of STAT6, Akt (ser473), and ERK in BMDMs treated with GH3 or MMQ cell-derived CM, M-CSF (10 ng/ml), or IL4 (10 ng/ml) for 15 min. **D,** Concentration of lactic acid in CM from oxamate-treated or untreated PA cells. **E,** Quantification of *Cd301* and *Arg1* mRNA expression in BMDMs stimulated with lactic acid/oxamate alone, or CM from oxamate-treated PA cells or CM from PA cells for 24 h.** F,** Western blot analysis of Akt (ser473) and ERK in BMDMs stimulated with GH3 or MMQ cell-derived CM or CM from oxamate-treated GH3 and MMQ cells for 15 min.** G,** Quantification of *CD301* and *ARG1* mRNA expression in THP1 cells treated with lactic acid at indicated concentration for 24 h. **H,** Western blot analysis of Akt (ser473) and ERK in THP1 cells treated with lactic acid at indicated concentration for 15 min. **I**, Representative images showing tumors harvested from mice bearing tumors in the GH3, GH3+LA, GH3+M0, and GH3+M0+LA group. **J,** Weight of mice bearing tumors in the GH3, GH3+LA, GH3+M0, and GH3+M0+LA groups. **K,** Stacking bar chart of the number of infiltrated CD11b^+^/F4/80^+^ and CD11b^+^/F4/80^+^/CD206^+^/CD11c^-^ cells in harvested tumors from the GH3, GH3+LA, GH3+M0, and GH3+M0+LA groups. **L,** Western blot analysis of Akt (ser473), and ERK in tumor tissues from mice in the GH3, GH3+M0, and GH3+M0+LA groups. *P < 0.05, **P < 0.01, and ***P < 0.001.

Lactate has recently been reported to play a role in modulating immune cells in the TME 23. Lactate favors the repolarization of M1-like macrophages towards the immunosuppressive M2-like state, which promotes the release of trophic factors, metabolic modulators, and immunosuppressive molecules 24. To elucidate whether lactate-mediated promotion of malignant behaviors in PA requires the support of TAMs, we used CM from GH3 and MMQ cells to treat rat bone marrow-derived macrophages (BMDMs). Unstimulated or unactivated BMDMs were regarded as M0 (resting macrophages). The concentration of lactic acid in CM was measured and presented as a lactate gradient (2.75, 5.15, and 8.15 mmol/L in GH3-derived CM and 3.95, 7.15, and 9.1 mmol/L in MMQ-derived CM, respectively, *versus* 0.245 mmol/L in primary medium) (Figure 3A). After 24 h, we performed qPCR and immunofluorescence staining and found that the expression levels of M2 macrophage markers, especially CD301 and ARG1, were dramatically increased in BMDMs treated with PA cell supernatant compared with those in BMDMs treated with the respective control media (Figure 3B and Figure S4).

Previous studies have illustrated that M2 macrophages are maintained mainly by IL4-STAT6 and MCSF-mTORC2 signaling 25, 26. To assess the mechanism by which lactate regulates the M2 macrophage population, we exposed macrophages to CM and measured that the phosphorylation levels of the mTORC2 downstream targets Akt (Ser473) and ERK but not of STAT6 signaling in a dose-dependent manner (Figure 3C). To verify that lactate is essential in this process, we treated GH3 cells with sodium oxamate, an LDHA inhibitor, to reduce lactic acid production in CM (Figure 3D), and we treated BMDMs with lactic acid or oxamate alone, as positive or negative control, respectively. The lower levels of lactic acid in the culture media failed to induce the polarization of macrophages to a M2-like state and inhibited the activation of the mTORC2 pathway (Figure 3E-F). Furthermore, we treated THP1 cells with the indicated concentrations of lactate and found that the expression levels of M2-related genes (CD301 and ARG1) significantly increased in response to 8 mmol/L lactate and that lactate-induced phosphorylation of Akt (Ser473) and the ERK pathway showed significant activity in a dose-dependent manner, suggesting that these two pathways are potentially involved in the lactate-induced polarization of M2-like TAMs (Figure 3G-H).

To clarify the effect of lactic acid *in vivo*, we injected NOD/SCID mice with either an admixture of GH3 cells and BMDMs or GH3 cells alone. After 30 days when the tumor was palpable and reached a volume of approximately 62.5 mm^3^, we injected lactic acid into the tumor. As shown in Figure 3I and 3J, a statistically significant increase in size (3.17-fold, P < 0.0001 *versus* GH3 control; 2.44-fold, P < 0.0001* versus* GH3 + M0 group) was observed in tumors stimulated with lactic acid. However, there was no significant difference in size among the control group, GH3+LA group, and GH3 + M0 group. The results of low cytometry indicated that lactic acid plays a critical role in inducing the polarization of TAMs toward the M2 phenotype (CD11b^+^/F4/80^+^/CD206^+^/CD11c^-^) and shifting the activity of TAMs to exert protumor effects (Figure 3K). In addition, the lactic acid-treated group showed significant activation of the mTORC2 downstream pathway and ERK pathway compared with that of the control and untreated groups (Figure 3L).

These results demonstrated that overproduction of lactate derived from invasive PAs shifts TAMs towards the M2 phenotype *in vitro* and *in vivo*, resulting in remodeling of the TME of PAs.

### CCL17 derived from lactate-activated TAMs mediates the invasion of PAs

**Figure 4:**
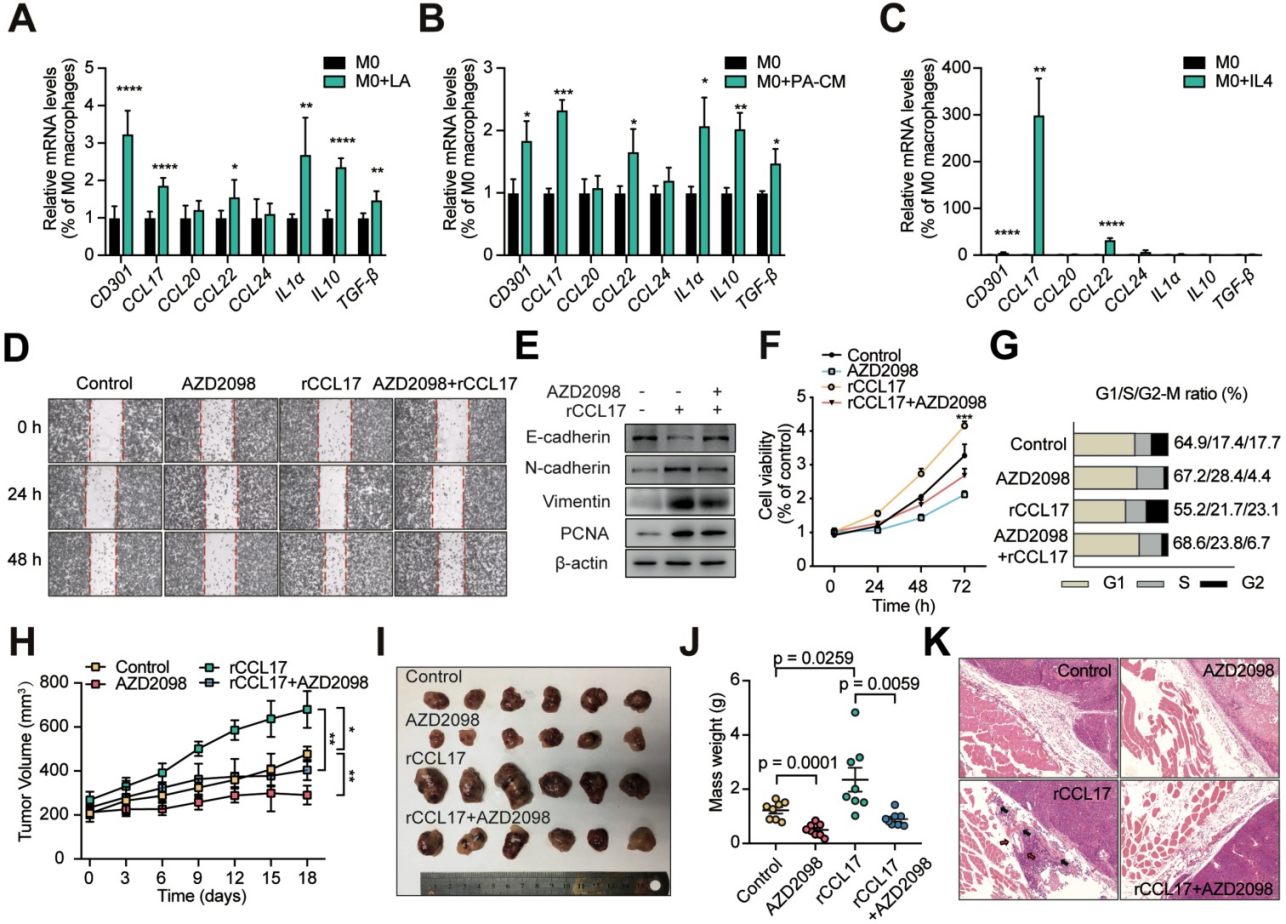
** CCL17 derived from lactate-activated TAMs mediates the invasion of PAs through binding to CCR4. A-C,** Quantification of various TAM-derived chemokines and cytokines in PMA-treated THP1 cells stimulated with lactic acid (**A**), CM from human primary PA cells (**B**), or IL4 (10 ng/ml) (**C**) for 24 h.** D,** Representative images of wound-healing assay using GH3 cells in the presence or absence of CCL17 (50 ng/ml), AZD2098 (20 µM), or combination. **E,** PCNA and EMT biomarker protein expression in GH3 cells under stimulation with CCL17, AZD2098, or combination for 24 h.** F,** Proliferation of GH3 cells following 24, 48, and 72 h stimulation under CCL17, AZD2098, or combination. **G,** Cell cycle assays in GH3 cells in the presence or absence of CCL17, AZD2098, or combination. **H,** Tumor growth curve in tumors derived from NOD/SCID tumor-bearing mice treated with PBS, AZD2098 (1.5 mg/kg), CCL17 (0.1 µg/kg), or combination. **I,** Representative images and **J,** mass weight of tumors derived from NOD/SCID tumor-bearing mice treated with PBS, AZD2098, CCL17, or combination. **K,** HE staining of edge region of tumor and muscle tissues derived from the PBS, AZD2098, CCL17, or combination group. All t-tests were two-tailed. Mean ± SEM. *P < 0.05, **P < 0.01, and ***P < 0.001.

We next investigated the mechanism of lactate-induced M2-like TAMs enhancement of proliferation and invasion of PAs. To explore the mechanisms by which M2-like TAMs impact PA invasion, we assessed the expression levels of three cytokines (IL1α, IL10, and TGF-β) and four chemokines (CCL17, CCL20, CCL22, and CCL24) derived from TAMs 13, 14, 27, 28. RT-qPCR was performed to estimate the mRNA expression levels of these seven cytokines as well as of *CD301* in THP1 cells treated with lactic acid, CM from primary human PA cells, and IL4. CCL17 and CCL22 expression levels in THP1 cells were markedly different in response to the multiple M2-like phenotype-inducing conditions compared with the control treatments (Figure 4A-C). Based on these results, the chemokines CCL17 and CCL22 chemokines were selected as potentially relevant cytokines.

To explore whether CCL17 and/or CCL22 promotes tumor invasion, recombinant rat CCL17 (rCCL17) and CCL22 (rCCL22) proteins at a given concentration were used to stimulate GH3 cells (Figure S5A). The rCCL17-treated group showed significantly enhanced cell migration compared with that of the control and rCCL22-treated groups (Figure S6A). Furthermore, EMT phenotypes were markedly observed in GH3 cells treated with rCCL17 for 24 h as indicated by decreased E-cadherin expression and increased N-cadherin, Vimentin, and PCNA expression (Figure S6B). In addition, rCCL17 significantly promoted cell proliferation (Supplementary Fig. 6C and D). However, CCL22 showed no promoting effects on the migration, invasion or proliferation of GH3 cells (Figure S6). In contrast, CCL17 expression was greatly increased in tumor tissues from mice in the GH3+M0+LA group, similar to LDHA and Lamp2 levels, compared with samples from the control and untreated groups (Figure S7A-C). Moreover, a positive correlation between the expression level of CCL17 and tumor size was observed in tumor-bearing NOD/SCID mice (Figure S7D), which demonstrated the promoting function of M2-like TAM-derived CCL17 in invasive PAs.

Together, the above results suggested that M2-like TAM-derived CCL17 facilitates the invasion of PAs.

### CCL17 promotes PA invasion by binding to CCR4

It has been previously reported that blocking CCR4, the CCL17 receptor, inhibits tumor growth and prolongs survival 29. To elucidate whether CCL17 promotes cell proliferation and migration by binding to CCR4, we added the CCR4 antagonist AZD2098 at a given concentration (Figure S5B), which markedly suppressed the promoting effects of CCL17 on cell migration, invasion and proliferation (Figure 4D-G). Next, to investigate the effect of the CCL17/CCR4 axis on PA invasion *in vivo*, a tumor-bearing NOD/SCID mouse model was utilized. At day 40 when the tumor reached a volume of 100-200 mm^3^, mice were treated with CCL17 (250 ng/kg), AZD2098 (1.0 mg/kg), CCL17+AZD2098, or PBS (vehicle control) every two days, and tumor sizes were measured. At day 44, the mice were sacrificed. Both the volumes and weights of the resulting tumors were higher in the CCL17 injection group than in the control group. In contrast, treatment with AZD2098 markedly reduced the CCL17-induced tumor burden (Figure 4H-L). Moreover, HE staining indicated that intratumoral injection of rCCL17 enhanced the vascular invasion and penetration of GH3 cells into adjacent normal tissues compared with those of the control group, whereas treatment with AZD2098 markedly decreased these effects (Figure 4K). Similarly, these malignant biological properties were significantly reversed by treatment with a CCR4 inhibitor. These findings demonstrate that CCL17 mediates PA invasion by binding to CCR4.

### The CCL17/CCR4 axis promotes tumor invasion *via* the mTORC1 signaling pathway

**Figure 5:**
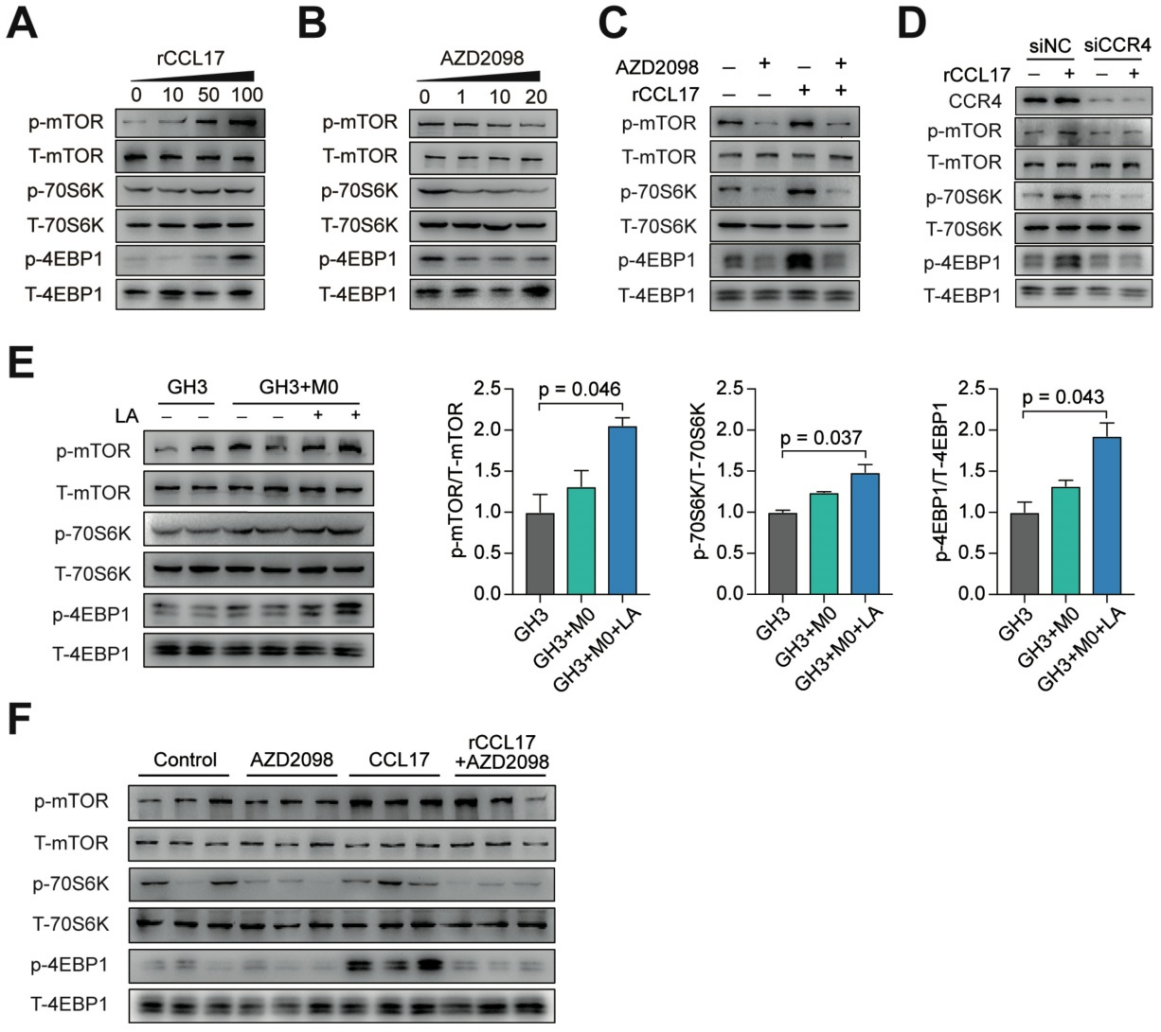
** The CCL17/CCR4 axis promotes tumor invasion *via* the mTORC1 signaling pathway. A and B,** Phosphorylation level of mTOR, 4EBP1, and 70S6K in GH3 cells following treatment with the indicated concentration of CCL17 (**A**) or AZD2098 (**B**) for 30 min. **C,** Western blot analysis of the activation of the mTORC1 signal pathway in GH3 cells with or without exposure to CCL17 (50 ng/ml) following co-treatment with AZD2098 (20 µmol/ml) for 30 min. **D,** Western blot analysis of the phosphorylation level of mTOR, 4EBP1, and 70S6K in GH3 cells transfected with control siRNA (siNC) or CCR4 siRNA (siCCR4) followed by treatment with or without rCCL17, respectively, for 30 min. **E,** Representative and quantification of the Western blot analysis of the phosphorylation levels of mTOR, 4EBP1, and 70S6K in in tumor tissues from mice in the GH3, GH3+M0, and GH3+M0+LA groups.** F,** Western blot analysis of the phosphorylation level of mTOR, 4EBP1, and 70S6K in tumor tissues from NOD/SCID tumor-bearing mice treated with PBS, AZD2098, CCL17, or combination. All t-tests were two-tailed. Mean ± SEM.

Based on our *in vitro* and *in vivo* findings, we next sought to identify the potential downstream targets of CCL17/CCR4 activation. We analyzed publicly available data from the GSE22812 dataset and observed that the mTOR signaling pathway was significantly activated in 6 invasive PA tumors compared with 5 samples from noninvasive PAs (Figure S8). The mTORC1 signaling pathway was significantly activated in GH3 cells treated with rCCL17 in a dose-dependent manner, whereas AZD2098 showed the opposite effect with the same kinetics, suggesting that the mTORC1 pathway is potentially involved in the protumorigenic effects of CCL17 (Figure 5A-B). For further verification, GH3 cells were treated with a CCR4 antagonist, or CCR4 expression was downregulated in GH3 cells. As shown in Figure 5C and 5D, both AZD2098 treatment and CCR4 knockdown inactivated the mTORC1 pathway in rCCL17-treated GH3 cells. Moreover, we demonstrated that the mTORC1 pathway was significantly activated in the tumor samples from the GH3+M0+LA group and rCCL17-treated GH3 xenografted mice; injection with AZD2098 profoundly suppressed this activity (Figure 5E-F).

Taken together, these results demonstrated that CCL17 secreted by M2-like TAMs promotes tumor invasion by activating the mTORC1 signaling pathway.

### CCL17 is positively associated with tumor invasion and postoperative recurrence in human PAs

**Figure 6:**
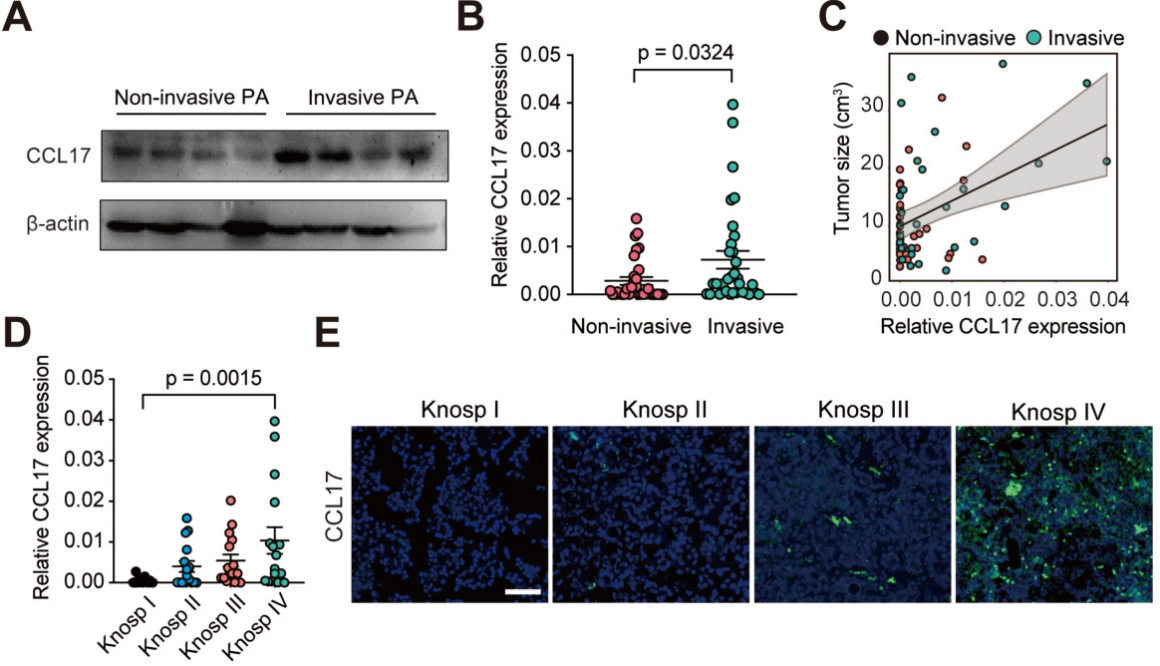
** CCL17 is positively associated with tumor progression in human PAs. A,** Western blot analysis of CCL17 expression in non-invasive PAs versus invasive PAs. **B,** *CCL17* mRNA expression levels in the non-invasive group (n = 32) and invasive group (n = 32). **C,** *CCL17* expression correlated with tumor size in samples from PA patients (n = 64). **D and E,** mRNA expression (**D**) and immunofluorescence (**E**) of CCL17 in human PA tissue samples according to Knosp grade classification. Original magnification, ×200. Scale bars, 50 µm. All t-tests were two-tailed. Mean ± SEM.

To better understand the role of CCL17 in human PAs, we measured CCL17 expression in PA tissues (*n* = 64) from patients who underwent surgical resection. We found that CCL17 expression was higher in invasive PAs than in noninvasive PAs at both the mRNA and protein levels (Figure 6A-B). Moreover, CCL17 levels positively promoted tumor growth in human PAs (Figure 6C). Consistent with this, when the patients were subdivided into 4 groups based on the Knosp classification system, CCL17 expression was higher in high-grade PA tissues than in low-grade tumor tissues as assessed by qPCR and immunofluorescence staining (Figure 6D-E).

To further investigate the clinical value of CCL17 in human PAs, we divided 64 patients into a high CCL17 expression group and a low CCL17 expression group according to CCL17 mRNA levels. As shown in Table 1, higher CCL17 expression predicted larger tumor size, greater invasion, and greater susceptibility to postoperative recurrence. Moreover, CCL17 expression was only associated with M2-like TAMs but not age, sex, or infiltration of M1-like TAMs, CD4^+^ T lymphocytes, CD8^+^ T lymphocytes and neutrophils.

**Figure 7:**
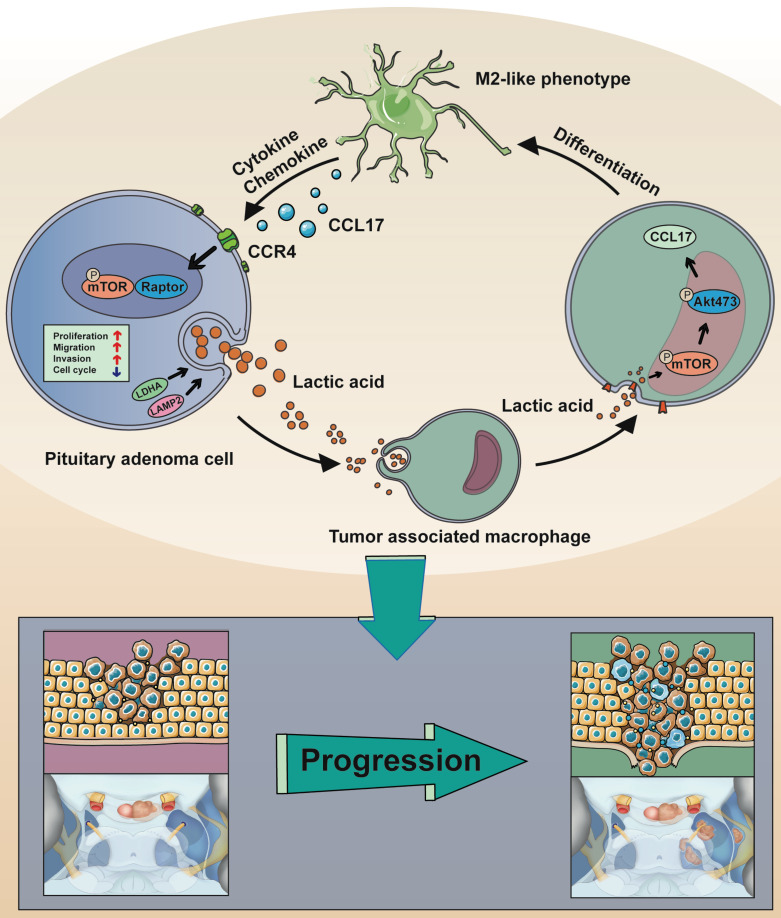
Schematic model showing the mechanism of lactate-mediated crosstalk between PA cells and tumor associated macrophages promoting tumor invasion. Overproduction of lactate in PA cells may contribute to malignant-like transformation at the early stage of PA formation. Lactate secreted by PA cells promote TAMs in the microenvironment towards M2 phenotype polarization *via* the mTORC2 and ERK1/2 pathways. In return, the CCL17 chemokine secreted by lactate-induced M2-like TAMs binds to its cognate receptor CCR4 on PA cells, thereby activating PA cells proliferation and invasion *via* the mTORC1 pathway.

Taken together, these data (Figure 7) demonstrated that CCL17 secretion from lactate-induced M2-like TAMs is linked with the tumor invasion of human PAs.

## Discussion

Previous studies have focused on genome function and the tumor itself to illustrate the mechanisms of tumor invasion in PAs 30. The reciprocal regulation between tumor cells and stromal cells shapes the status of the TME, which may determine tumor outcomes and may be a novel mechanism that influences tumor invasion. Here, we demonstrated that overproduction of lactate in invasive PAs shifted TAMS toward an M2-like phenotype. Subsequently, these TAMs then secreted CCL17, which promoted tumor invasion *in vitro* and *in vivo via* activation of mTORC1 signaling. Clinical data showed that CCL17 expression is positively correlated with tumor invasion in PA patients.

Lactate has been shown to alter the transcriptional activity of several key oncogenes, and increased carcinogenesis signaling due to augmented lactate production can serve as one essential purpose of the Warburg effect 31. Lactate metabolism is particularly relevant for not only the metabolic equilibrium between hypoxic (lactate-generating) and normoxic (lactate-importing) cancer cells 32 but also the polarization of TAMs by hypoxic cancer cells toward a poorly glycolytic M2-like profile 33-35. We discovered that the function of PA cell-derived lactate on TAM polarization may be the first step to enhance PA invasion. Of note, M2 polarization of melanoma-associated TAMs is promoted by sensing acidification of the TME, which is induced by increased glycolysis in cancer cells 36. Changes in extracellular lactate have been demonstrated to affect cells from both the lymphoid and myeloid lineages. Lactate specifically biases TAMs towards a “tumor-friendly” M2-like phenotype, which contributes to tumor cells evading immunosurveillance 37. In our study, we discovered that invasive PAs overproduced lactate to promote TAM polarization toward a M2-like phenotype. These data indicated that lactate plays a critical role in remodeling the TME and regulating the intracellular crosstalk between PA cells and TAMs.

TAMs are key regulators of homeostasis in tissues and the TME. TAMs in the primary tumor promote tumor invasion and metastasis, and they are regarded as an attractive target as part of combination therapies in cancer treatment 8, 9, 38. In the 1990s, immune cell infiltration in PAs was first reported based on the presence of a low number of macrophages as well as CD8^+^ and CD4^+^ lymphocytes in close proximity to the tumors 10. Recently, studies have revealed a correlation between CD68^+^ macrophage infiltration and PA invasion, and the number of M2 macrophages is greatly increased in rats with diethylstilbestrol (DES)-induced PRL 11. However, the specific function of TAMs in PAs has not been elucidated. In the present study, we discovered that macrophages infiltrated PAs and dominated the population of immune cells, similar to the results of previous studies 10, 11. Furthermore, infiltration of M2-like TAMs was positively correlated with tumor invasion in SAs, PRLs and NFPAs but not in ACTH adenomas, suggesting that the lactate-induced changes in the immune cell population within the TME is the second step to enhance tumor invasion.

TAMs can contribute to tumor invasion by promoting tumor cell proliferation, EMT, and invasion as well as suppressing tumoricidal immune cells 8, 39, 40. The important role of TAMs in pituitary tumors has also been highlighted in two other studies 41, 42. A variety of cytokines, chemokines, and other immune mediators is secreted by macrophages during immune responses, which serves as a means of intracellular communication 43. Zhou et al. reported that the CCL18 chemokine secreted from M2 macrophages promotes migration and invasion in gallbladder cancer *via* the PI3K/Akt pathway 44. Wang et al. demonstrated that high miR-100 levels maintain the M2-like phenotype of TAMs and promote tumor metastasis by enhancing IL-1ra secretion 45.

CCL17 and its receptor CCR4 have been shown to exert a significant influence on homeostasis and inflammatory responses 46. Our results illustrated that CCL17 secreted from M2-like TAMs significantly increased the proliferation and invasion of PAs. Conversely, blocking CCR4 in GH3 xenograft mice significantly inhibited tumor growth and the malignant biological properties of the tumors. Thus, antagonizing CCR4 signaling is a potential treatment approach for PAs 47.

## Conclusions

In the present study, we illustrated for the first time the crosstalk between tumor cells and the TME in PAs. PAs can overproduce lactate, leading to TME acidification, which remodels TAMs toward an M2-like phenotype as the first step to promote PA invasion. Subsequently, these TAMs then secrete CCL17 to enhance tumor invasion *via* the CCL17/CCR4/mTORC1 axis. PAs with greater invasive properties tended to produce more lactate, which leads to an adaptive protumoral TME. Moreover, we illustrated that higher CCL17 expression in human PAs predicts larger tumor size, greater invasion, and more susceptibility to postoperative recurrence than PAs with lower CCL17 expression, thereby emphasizing the clinical value of CCL17 as a prognostic marker.

## Supplementary Material

Supplementary figures and tables.

## Figures and Tables

**Table 1:** Correlation of CCL17 expression levels with clinical pathological status in 64 cases of patients with PAs

*CCL17* expression levels	Low level	High level	*P* value
**Age (years)**			
≤45	15	18	0.453
>45	17	14	
**Gender**			
Male	16	14	0.6164
Female	16	18	
**Tumor Size (cm)**			
≤2	22	14	0.0438*
>2	10	18	
**Invasive Phenotype**			
No	21	12	0.0244*
Yes	11	20	
**Knosp Grade**			
I	15	5	0.0334*
II	6	7	
III	6	7	
IV	5	13	
**Recurrence**			
No	26	18	0.0195*
Yes	4	12	
**M2-like TAM Infiltration**			
Low	22	10	0.0027**
High	10	22	
**M1-like TAM Infiltration**			
Low	14	16	0.501
High	18	16	
**CD4^+^ T Lymphocytes Infiltration**			
Low	18	13	0.2111
High	14	19	
**CD8^+^ T Lymphocytes Infiltration**			
Low	19	15	0.3164
High	13	17	
**Neutrophil Infiltration**			
Low	17	16	0.8025
High	15	16	

## Abbreviations

PA: pituitary adenoma
TME: tumor microenvironment
TAM: tumor-associated macrophage
CCL17: C-C motif chemokine 17
CCR4: chemokine receptor 4
mTOR: mammalian target of rapamycin
HCC: hepatocellular carcinoma
MRI: magnetic resonance imaging
MPO: myeloperoxidase
PBS: phosphate-buffered saline
DMSO: dimethyl sulfoxide
CM: conditioned medium
LA: lactic acid
EMT: epithelial-mesenchymal transition
WHO: World Health Organization
HE: hematoxylin and eosin
IHC: immunohistochemical
DAPI: 4,6-diamidino-2-phenylindole dihydrochloride
GEO: Gene Expression Omnibus
GSEA: Gene Set Enrichment Analysis
LDH: lactate dehydrogenase
LAMP2: lysosome-associated membrane protein 2
SA: somatotroph adenoma
PRL: prolactinoma
NFPA: nonfunctioning pituitary adenoma
ACTH: adrenocorticotropic hormone
BMDM: bone marrow-derived macrophage
