# Supplementary material for: Lactate-induced M2 polarization of tumor-associated macrophages promotes the invasion of pituitary adenoma by secreting CCL17
Source: Theranostics. 2021 Feb 6;11(8):3839–52. doi: 10.7150/thno.53749 (PMC7914368; doi:10.7150/thno.53749)
Supplement: Supplementary file 1 — Supplementary figures and tables. [file thnov11p3839s1.pdf]

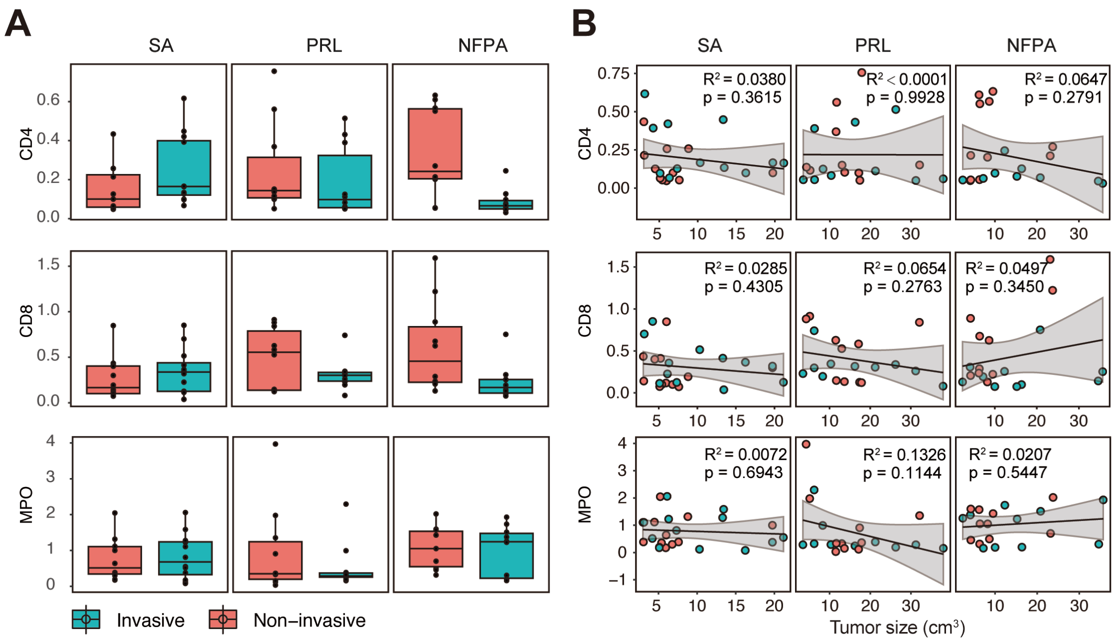

Supplementary Figure 1. Additional correlation between tumor characteristics and immune infiltration. A, Difference of CD4, CD8 or MPO levels in invasive PAs and noninvasive PAs in SA, PRL, and NFPA. **B,** Correlation of tumor size and relative expression of CD4, CD8 or MPO in SAs, PRLs, and NFPAs. Each dot represents the value of a patient. Mean ± SEM.

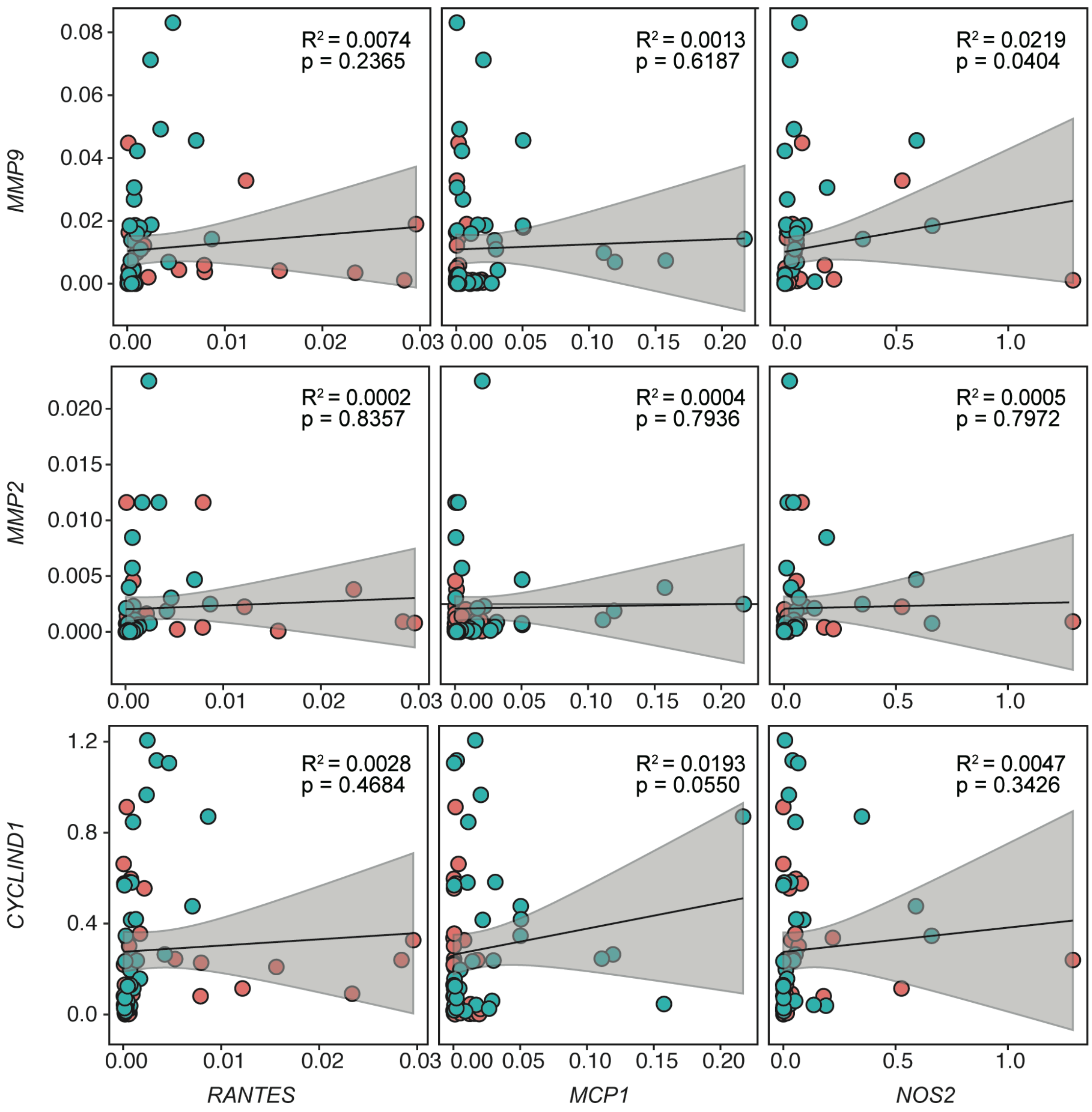

Supplementary Figure 2. Correlation between tumor invasion -related genes and M1 macrophage biomarkers. Correlation of M1 macrophage biomarkers and *MMP2*, *MMP9*, and *CYCLIND1* in tumor samples from PA patients. Each dot represents the value of a patient.

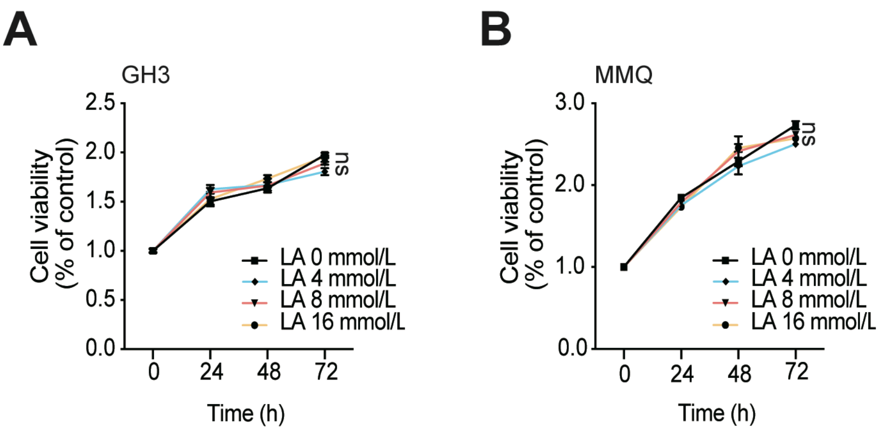

Supplementary Figure 3. Lactate did not directly enhanced PA cell proliferation. A and B, Cell proliferation assay of (**A**) GH3 and (**B**) MMQ cells in the present or absent of increasing concentration of lactic acid for 24, 48, and 72 h. NS, no significance.

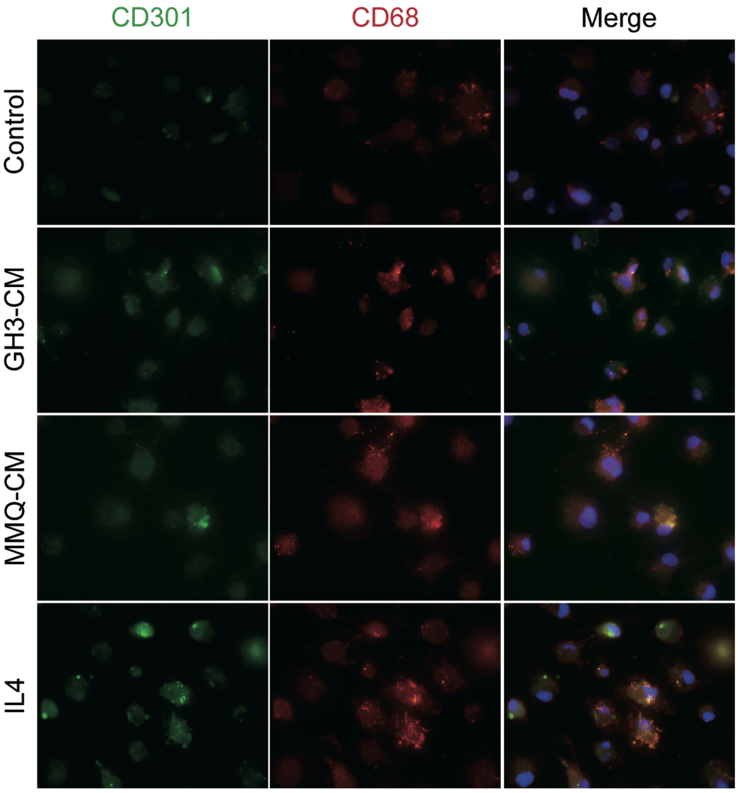

Supplementary Figure 4. immunofluorescence images of BMDMs. Representative immunofluorescence images of BMDM co-stained with CD301 (green) and CD68 (red) following stimulation with the PBS, CM, or IL4 (10 ng/ml) for 24 h. Cell nuclei were counterstained with DAPI.

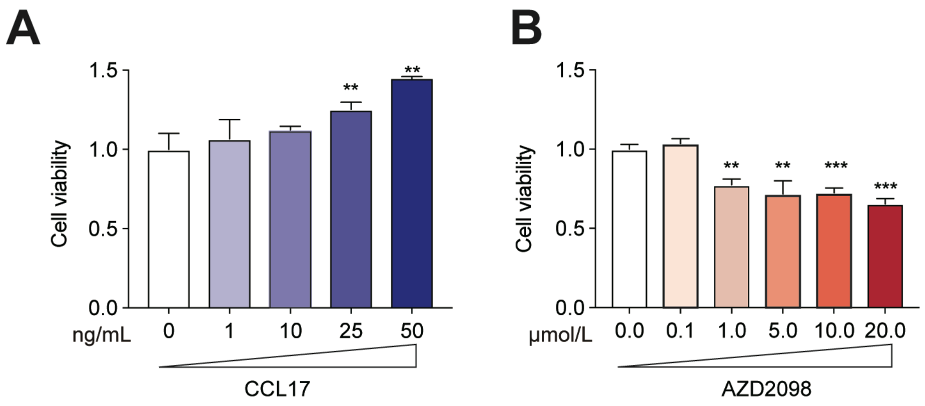

Supplementary Figure 5. Selection of stimulus concentration gradient. A and B, Cell viability of GH3 treated with the indicated concentration of (**A**) rCCL17 or (**B**) AZD2098 for 24 h. Statistical significance was determined using *one-way ANOVA*. *P < 0.05, **P < 0.01, and ***P < 0.001.

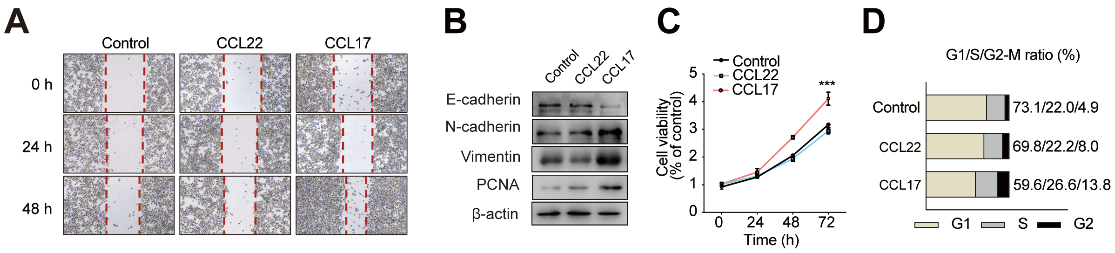

Supplementary Figure 6. CCL17 promoted cell proliferation and invasion of PA cells. A, Representative images of wound scratching assay of GH3 cells following treatment of PBS, rCCL22, or rCCL17 for 24 and 48 h. **B,** PCNA and EMT biomarkers protein expression in GH3 cells under stimulation of PBS, rCCL22, or rCCL17 down-regulated TAMs for 24 h. **C,** Proliferation of GH3 cells following stimulation with PBS, rCCL22, or rCCL17 for 24, 48, and 72 h. **D,** Cell cycle assays in GH3 cells in the presence or absence of rCCL22, or rCCL17 for 24 h. All t-tests were two-tailed. Mean ± SEM. ***$P < 0.001$.

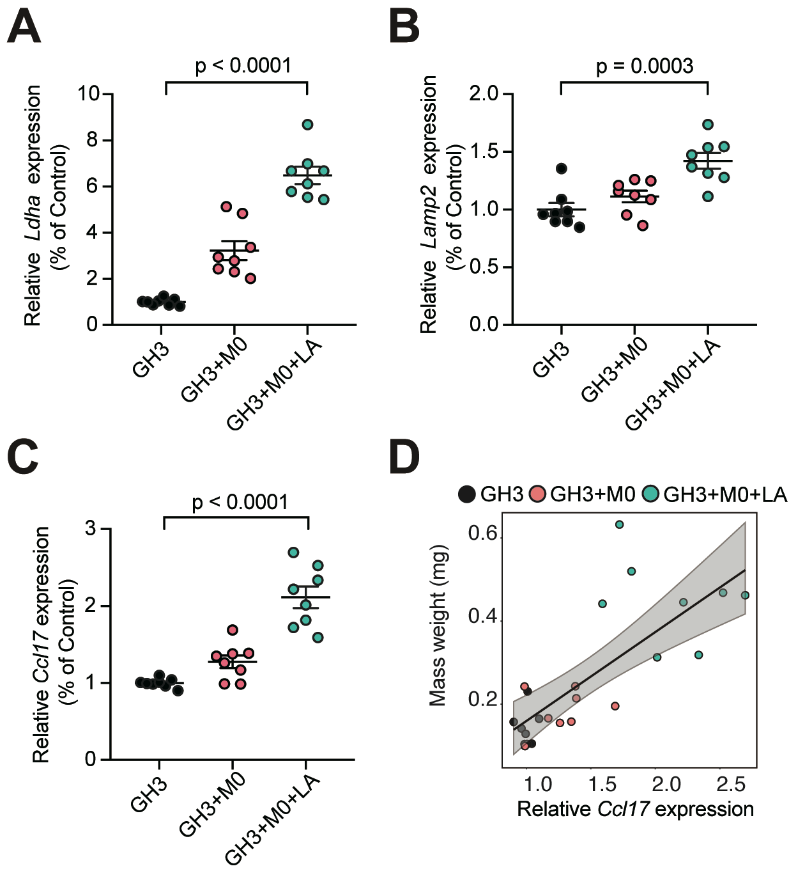

Supplementary Figure 7. Tumor characteristics and CCL17 expression in tumor bearing Nod Scid models. A-C, mRNA expression of **(A)** *LDHA*, **(B)** *LAMP2*, and **(C)** *CCL17* in tumors from GH3, GH3+M0, and GH3+M0+LA group (n = 8). **D,** Positive correlation between *CCL17* levels and mass weight of tumors from GH3, GH3+M0, and GH3+M0+LA group (n = 8). All t-tests were two-tailed. Mean ± SEM.

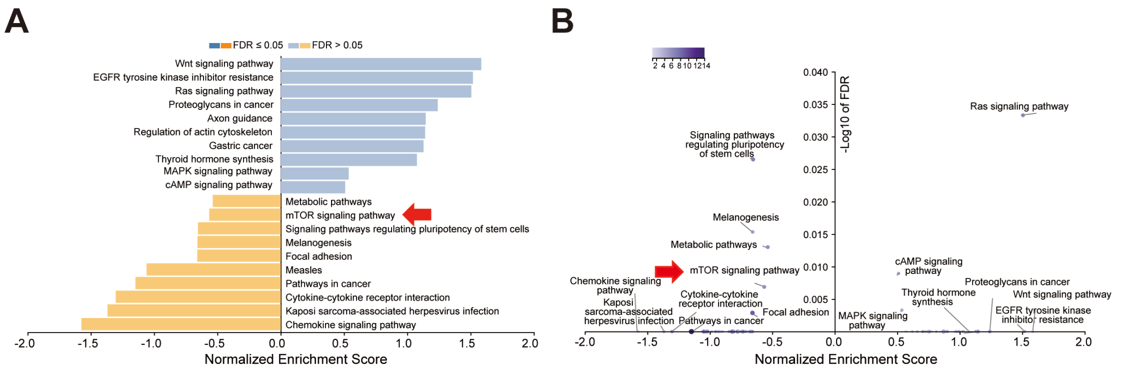

Supplementary Figure 8. Bioinformatic analysis of invasive and non-invasive PAs RNA-seq profile. A and B, The customizable volcano plot **(A)** and bar chart (**B**) show enrichment ration or NES of results with the direction indicated the role of mTOR signal pathway in the progression of pituitary adenoma.

Supplementary Table 1. PCR primers.

Genes	Primer-forward	Primer-reverse
**Human**		
CYCLIND1	GCCGAGAAGCTGTGCATCTA	GAAATCGTGCGGGGTCATTG
MMP9	GTACTCGACCTGTACCAGCG	AGAAGCCCCACTTCTTGTCG
MMP2	AACCAGCTGGCCTAGTGATG	CTTGGGGCAGCCATAGAAGG
CD206	GCAGAAGGAGTAACCCACCC	ATTTGGGTTCGGGAGTCGTC
CD301	GCCAGGTGGCTACTCTCAAC	TAGCAGCTGTCTTGGTGCTC
ARG1	ACTTAAAGAACAAGAGTGTGATGTG	ATTGCCAAACTGTGGTCTCC
RANTES	ATGACTCCCGGCTGAACAAG	CACACTTGGCGGTTCTTTCG
MCP1	AGAGGCTGAGACTAACCCAGA	TTTCATGCTGGAGGCGAGAG
NOS2	CGCATGACCTTGGTGTTTGG	CATAGACCTTGGGCTTGCCA
CCL17	GGACGAAGAAGAGCCACAGT	GCTCCAGTTCAGACAAGGGG
LDHA	AGCTGTTCCACTTAAGGCCC	AATGAGATCCGGAATCGGCG
LAMP2	CGCCGATTCCTGGCTTTTG	AAGAGCACTGATGACCACCG
ACTB	CTCACCATGGATGATGATATCGC	CCACATAGGAATCCTTCTGACC
**Rat**		
Cd301	AGATGAAGCTGGCCAAGGAC	GAGGAGTCCAAACTCCAGGC
Arg1	GGACATCGTGTACATCGGCT	CTTCCTTCCCAGCAGGTAGC
Mcp1	TAGCATCCACGTGCTGTCTC	TTCTCCAGCCGACTCATTGG
Nos2	ACACAGTGTCGCTGGTTTGA	ACCAACTCTGCTGTTCTCCG
Ccl17	GCCTGGACTACTTCAAGGGG	CACTGAGGTCCTGAACCACG
Ldha	TAGCACTTCACTGTCCAGGC	AACACAACTGGACCAACTGGA
Lamp2	AGCCCTGGGAGGAGTACTTA	TGATGGCGCTTGAGACCAAT
Actb	GCAGGAGTACGATGAGTCCG	ACGCAGCTCAGTAACAGTCC
